# A study on the fabrication of metal microneedle array electrodes for ECG detection based on low melting point Bi–In–Sn alloys

**DOI:** 10.1038/s41598-023-50472-y

**Published:** 2023-12-21

**Authors:** Hyunjong Gwak, Sungbo Cho, Yoon-Jae Song, Jung-Hwan Park, Soonmin Seo

**Affiliations:** 1 Department of BioNano Technology, Gachon University, Seongnam-Si, Gyeonggi-Do 13120 Republic of Korea; 2 Department of Electronic Engineering, Gachon University, Seongnam-Si, Gyeonggi-Do 13120 Republic of Korea; 3 Department of Life Science, Gachon University, Seongnam-Si, Gyeonggi-Do 13120 Republic of Korea

**Keywords:** Biomedical engineering, Metals and alloys

## Abstract

This study describes the fabrication and characteristics of microneedle array electrodes (MAEs) using Bismuth–Indium–Tin (Bi–In–Sn) alloys. The MAEs consist of 57 pyramid-shaped needles measuring 340 μm wide and 800 μm high. The fabrication process involved micromolding the alloys in a vacuum environment. Physical tests demonstrated that Bi–In–Sn MAEs have good mechanical strength, indicating their suitability for successful skin penetration. The electrode–skin interface impedance test confirmed that Bi–In–Sn MAEs successfully penetrated the skin. Impedance measurements revealed the importance of insulating the microneedle electrodes for optimal electrical performance, and a UV-curable Polyurethane Acrylate coating was applied to enhance insulation. Electrocardiogram measurements using the Bi–In–Sn MAEs demonstrated performance comparable to that of traditional Ag/AgCl electrodes, which shows promise for accurate data collection. Overall, the study demonstrates successful, minimally-invasive skin insertion, improved electrical insulation, and potential applications of Bi–In–Sn microneedle array. These findings contribute to advancements in microneedle technology for biomedical applications.

## Introduction

An electrocardiogram (ECG) records the active current and potential difference generated by the contraction of the heart, making it the best method for measuring and diagnosing abnormal heart rhythms. Numerous methods have been studied thus far^1–4^. Specifically, an ECG records the active electrical current starting from the SA node, a pacemaker cell, which moves inside the heart along the AV node, bundle of His, and Purkinje fiber, causing the heart to contract. This series of processes repeats continuously. Depending on the order in which the waveforms appear, they are divided into five components: P, Q, R, S, and T. By analyzing the abnormalities in each waveform, problems in the detailed position of the heart can be diagnosed. The waveforms may vary depending on the measurement method^5,6^.

**Figure 1:**
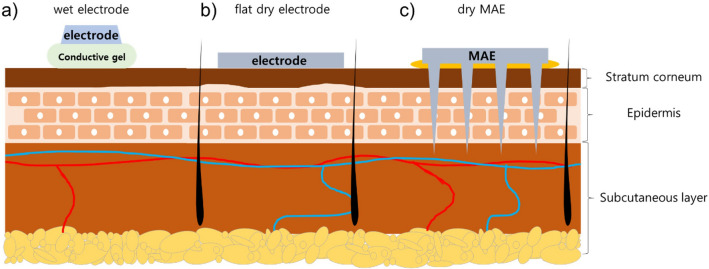
Images of interface between skin and (**a**) wet electrode with conductive gel, (**b**) flat dry electrode, and (**c**) MAE without conductive gel.

The measurement of an ECG involves the use of attachable electrodes to observe the electrical signals generated by the body. These electrodes can generally be categorized into two types: wet electrodes and dry electrodes^7^. As shown in Fig. 1a, wet electrodes typically use an Ag/AgCl electrode along with a conductive gel such as hydrogel, Polyethylene glycol (PEG), or Polypropylene glycol (PPG) to reduce the impedance of the stratum corneum. The Ag/AgCl electrode is preferred for wet electrodes due to its resistance to rust. However, while it provides clear signals, it requires pretreatment (such as hair removal and disinfection), can cause gel inflammation or allergic reactions, and cannot be used for extended periods due to the hardening of the gel over time^8^. As shown in Fig. 1b, dry electrodes utilize various metals such as gold, silver, and stainless steel. Flat dry electrodes have the disadvantage of poor skin contact, resulting in high impedance and difficulties in obtaining clear signals. Without a sweat layer serving as an electrolyte, clear signals are challenging to obtain, and adhesion to the skin is more difficult compared to wet electrodes. However, the advantage of dry electrodes is that they do not require pretreatment of the skin for signal measurement^9,10^.

Both types of electrodes measure the ECG through the skin. The stratum corneum, located on the outermost layer of the skin, consists of dead cells, resulting in high impedance that poses an obstacle to accurate ECG measurement. The impedance of the stratum corneum may vary depending on factors such as season, time, environment, and the presence or absence of an electrolyte layer such as sweat or conductive gel. Consequently, several studies are being conducted to determine how to minimize interference of the stratum corneum, which almost functions as an insulating layer^8,10^.

To overcome the challenges posed by the stratum corneum, a microneedle array electrode (MAE) was developed as a dry electrode^11^. MAEs consist of tens to hundreds of fine conductor needles, each with a length of hundreds of micrometers, arranged in an array formation. Microneedles have been developed to pass minimally-invasively through the stratum corneum to deliver drugs into the skin layer or to extract body fluid from the skin layer. In particular, microneedles have been developed to minimize the pain caused by insertion into the skin. Studies have shown that the Visual Analogue Scale (VAS) of pain caused by microneedles less than 1 mm in length is below 1, indicating almost no experience of pain^12^. MAEs serve as electrodes that can directly capture biosignals from the skin through the stratum corneum, as depicted in Fig. 1c.

To ensure conductivity, these electrodes are manufactured in the shape of cones or square cones using various materials, including metals, polymers coated with metals, or metals coated with polymers. In some cases, they are even designed as hollow microneedles. However, the production of MAEs using metal-based materials can be challenging because of the inherent difficulty in molding complex structures^11,13–16^.

Typically, metals have melting points ranging from several hundred to more than 1000 °C. To create desired shapes using these metals, materials such as clay or sand, which are capable of withstanding high temperatures, are used to make master molds. However, when metals with melting points lower than 100 °C are used, materials such as polydimethylsiloxane (PDMS) or polymers that do not melt or decompose at lower temperatures can be used to make the master molds. This enables the fabrication of intricate metal patterns and the use of metals with low melting points for MAEs. Recently, a study has been presented on the development of non-reusable intravenous needles using Gallium (Ga) metal, known for its low melting point^17^. This research on needles utilizing such low-melting-point metals is expanding potential applications in the biomedical field. While researches on low-melting-point metals based on Ga are well-known, experimental findings on the characteristics and applications of Bi-based low-melting-point metals are not widely investigated^18^. Bi–In–Sn alloy (Field's metal), an alloy consisting of 32.5% bismuth, 51% indium, and 16.5% tin, has a melting point of 62 °C. Unlike metals such as Wood's metal, Rose's metal, and Cerrolow, which have low melting points, Field's metal does not contain lead or aluminum. Consequently, it is expected to be a suitable material for MAEs that can be attached to human skin. According to recent research, it has been reported that this Bi–In–Sn alloy material exhibits non-toxicity and very good biocompatibility^19^. Moreover, it exhibits excellent heat and electrical conductivity compared to general metals, leading to extensive research in fields such as electrode fabrication, solar power generation, and photocatalysis. It is particularly attracting attention in the field of nanoscience^20–22^.

In this study, ECGs were measured with MAEs manufactured using Bi–In–Sn alloy, which because of its relatively low melting point compared to other metals. Four considerations were taken into account in selecting this material. First, it is easier to manufacture MAEs using Bi–In–Sn alloy than existing MAEs. Second, Bi–In–Sn alloy can be integrated with the existing ECG measurement module. Third, MAEs with Bi–In–Sn alloy can maintain close contact with the skin. Finally, polymer coating can block elements unrelated to the heartbeat signal on the skin's surface and measure signals beneath the skin, thereby minimizing capacitance and electrical interference between the electrode and the skin layers.

To achieve these goals, we employed a molding method involving double-sided molding using two molds, and ECG measurements were conducted using this MAE. Compared to the previously available microneedle electrode, Bi–In–Sn alloy microneedles were manufactured using micromolding, a method used for mass production of dissolving microneedles for cosmetics. Using the established process for manufacturing commercial microneedles makes production of Bi–In–Sn alloy MAEs easy and economical, and the preparation of various shapes of these microneedles is possible by changing the microneedle mold.

Polyurethane Acrylate (PUA) was selected as the coating material. It is a photocurable substance that solidifies when exposed to ultraviolet (UV) light in its liquid state. It can be easily cured using UV light, has a low dielectric constant, and remains flexible even after curing, making it advantageous for skin adhesion^23,24^. Because of these characteristics, PUA is widely used in various fields, including the manufacture of stencil molds and in film production^25–28^. A PUA coating layer was used to reduce the contact between the outermost layer of the skin and the MAE.

## Methods

### Ethical statement

This study was approved by the IRB of Gachon University (IRB No. 1044396-202206-HR-115-01), written informed consent was obtained from the participant. And the authors declare that all methods were performed in accordance with the relevant guidelines and regulations.

### Fabrication of microneedle array electrode

**Figure 2:**
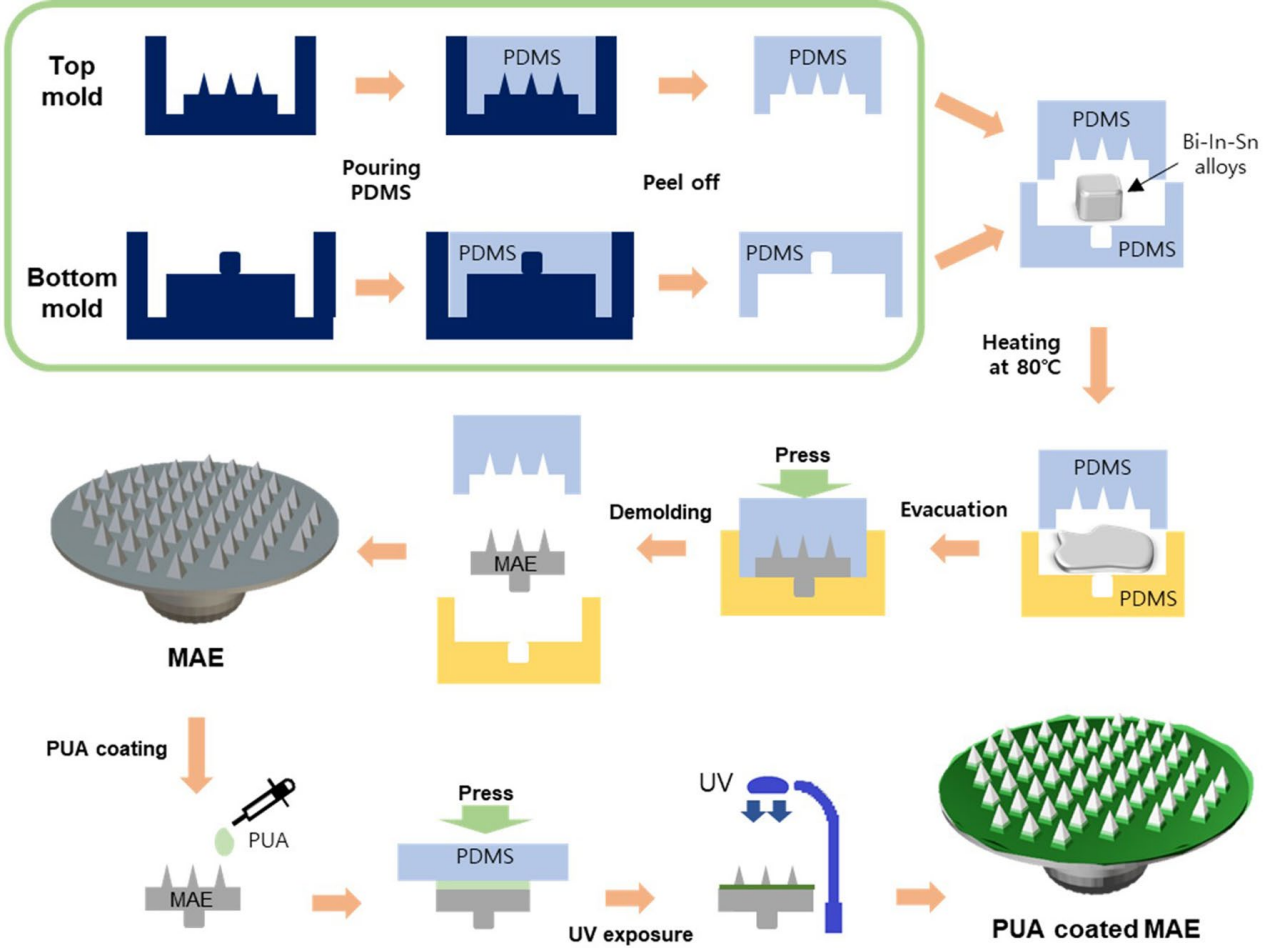
A schematic diagram of the fabrication process of the PUA-coated MAE through micromolding of Bi–In–Sn alloy.

Figure 2 shows a schematic diagram of the MAE fabrication process by micromolding of the Bi–In–Sn alloy. The master MAE mold, prepared using a micromilling technique, consists of 57 pyramid-shaped needles measuring 340 μm wide and 800 μm high. Two types of master molds were used: one for penetrating the skin, and the other for connecting the MAE to the ECG measurement system. Intaglio PDMS molds were replicated from these master molds. The top mold shown in Fig. 2 is an intaglio mold with 57 pyramid-shaped needles on the base, measuring 1 mm in height and covering an area of 1 cm^2^. The bottom mold has a bowl-shaped groove with a height of 3 mm and a diameter of 4 mm.

The MAEs were fabricated using a vacuum imprinting system. A lump of Bi–In–Sn alloy was placed on the bottom mold. After evacuating the vacuum chamber to below 1 torr, it was heated at 80 °C for 30 min and pressed with the top mold at a force of 15N. Excess Bi–In–Sn alloy was removed, and the sample was cooled down to 45 °C. Next, the top mold was removed and a 200 μm-thick PDMS film with a 1 cm diameter hole, serving as a guide for adjusting the PUA coating thickness, was placed on the bottom mold. A UV-curable PUA solution was dropped inside the hole and slightly pressed with a soft, flat PDMS. The pressed sample was exposed to UV light (λ ~ 365 nm) for 1 min to cure the PUA. After curing, the PDMS molds were peeled off. The manufactured PUA-coated Bi–In–Sn MAE was then coated with platinum, and the coating thickness, height, and width of the microneedle were measured using a scanning electron microscope (SEM, JSM-7500F, JEOL Ltd., Tokyo, Japan). And the thickness of coated PUA layer on base of microneedle array was measured using vernier calipers after removing the film layer.

### Physical test of MAE

The mechanical strength of the Bi–In–Sn MAE with PLA microneedles was measured using a force displacement machine (500N Zwicki, Zwick GmbH & Co. KG, Ulm, Germany)^29,30^. To ensure accurate comparison, each microneedle was measured by removing all but one of the 57 pyramid-shaped needles located at the center of the base. The MAE with one microneedle, measuring 340 μm wide and 800 μm high, was fixed upside down to the load cell of the force displacement machine. It was then pressed perpendicular to the microneedle at a speed of 5 mm/min.

To evaluate the ability of the MAEs to penetrate the skin, they were pressed into a full porcine skin (CRONEX, Seoul, Korea) at a force of 10N for 30 s and then removed. After the microneedles were removed, a 0.25% (v/v) trypan blue solution (Sigma-Aldrich, Seoul, Korea) was applied to the skin for 5 min to dye the penetrated area^31,32^. Any remaining trypan blue solution was then removed with phosphate-buffered saline (PBS) and a cotton swab. The holes were measured and counted using a stereo microscope (TL3000 Ergo, Leica Microsystems Ltd, Switzerland). It was determined that all the microneedles were successfully inserted into the skin layer. Thus, the Bi–In–Sn MAEs have sufficient mechanical strength to penetrate the stratum corneum, the main barrier to electrical signal detection.

### Measurement of electrochemical properties

To evaluate the electrical performance of the Bi–In–Sn MAEs, two measurements were performed using an impedance measurement machine (IVIUM Compactstat, potentiostat, IVIUM Technologies, Netherlands). First, the change in MAE impedance in a 0.9% (w/v) NaCl aqueous solution was measured according to PUA coating thicknesses (0 μm, 200 μm, 800 μm). Second, the impedance was measured at 50 mV amplitude in the frequency range of 0.1 Hz to 100,000 Hz, while varying the electrolyte concentration (0%, 0.001%, 0.01%, 0.1%, 1.0% [w/v] NaCl solution). Each measurement was performed five times for each sample, and the average value was calculated. The impedance measurement was conducted using a two-electrode method, with the electrode connected to the back surface of the Bi–In–Sn MAE serving as the working electrode and a platinum coil used as the counter electrode^13,31,32^. Since the experiment aimed to evaluate the insulation performance of the PUA coating, only the front surface of the microneedle was immersed in the solution.

### EII (electrode–skin interface impedance) test

Measurements were performed using force displacement machines (500N Zwicki, Zwick GmbH & Co. KG, Ulm, Germany) and Palmsens4 impedance measurement systems (PalmSens BV, Houten, Netherlands). A full porcine skin (CRONEX, Seoul, Korea) was prepared at the chuck of a force displacement machine, and the Bi–In–Sn MAE, which served as the working electrode, was designed to pass through the full porcine skin at a speed of 0.5 mm/s through copper wires attached upside down to the load cell. Ag/AgCl, a conventional wet electrode, was used as the counter electrode, and the distance between the two electrodes was fixed at 1 cm. Consequently, the change in impedance was measured according to the depth at which the Bi–In–Sn MAE penetrated the full porcine skin, and the force required to penetrate the stratum corneum of the full porcine skin and the electrical characteristics of the Bi–In–Sn MAE were evaluated. The frequency was fixed at 1000 Hz^11,13^.

### ECG measurement

An ECG was measured using a three-electrode method with the M4200 (RHS stim/recording controller, intan Technologies, Los Angeles, California), which is based on an integrated circuit capable of extracting, amplifying, and filtering small biosignals^11,13,14,33,34^. The ECG of a healthy male participant was measured by attaching an Ag/AgCl wet electrode and a Bi–In–Sn MAE to the wrists of both hands. An additional Ag/AgCl wet electrode was attached to the right ankle for grounding purposes. In this work, the electrical potential difference between the two wrists was recorded. To compare this potential difference, we kept all other environmental conditions the same except for the two measuring electrodes. Subsequently, the ECG was measured and compared for a duration of 1 min, starting from the time the ECG signal was stabilized. During the measurement, pressure was applied using a tegaderm patch to ensure stable electrode fixation.

## Results and discussion

### Observation of the shape of the microneedle electrodes

**Figure 3:**
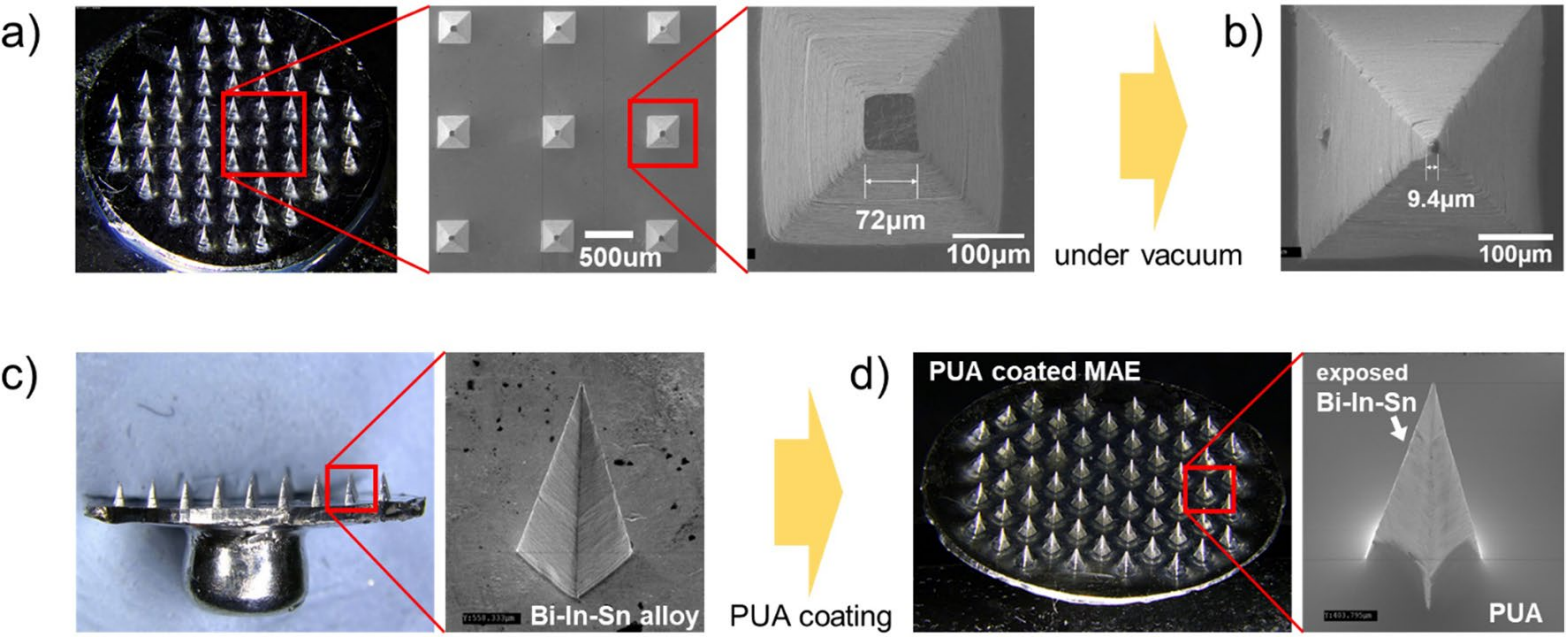
(**a**) Image of the fabricated Bi–In–Sn MAE after the micromolding process. The sharpness of the needle tip is around 72 μm when it is fabricated under atmospheric pressure, as shown in the magnified image. (**b**) After fabricating MAE in a vacuum environment, the sharpness of the needle tip decreases to below 10 μm. (**c**) Image of Bi–In–Sn MAE before PUA coating, along with its magnified SEM image. (**d**) Image of Bi–In–Sn MAE after PUA coating, along with its magnified SEM image.

Initially, the microneedles were fabricated using this process by heating the hot plate to 80 °C under atmospheric pressure and applying a force of 15N using a pneumatic press. After the process, the microneedles were allowed to cool to room temperature, followed by separation. Figure 3a shows that 57 microneedles are arranged on the base plate of a 1 cm^2^ MAE. Micromolding of Bi–In–Sn alloy is easily achievable by applying the appropriate temperature, pressure, and vacuum. The master mold has needles measuring 340 μm wide and 800 μm high. However, a fabricated microneedle at an atmospheric pressure has a height of less than 800 μm and a sharpness of over 10 μm, indicating that the micromolding process does not work smoothly for features less than 10 μm. Without using vacuum, the width of tip end of the microneedle is approximately 72 μm, which is not suitable for insertion through the skin. The partial replication of molten metal is caused by trapped air at the end of the PDMS mold and the trapped air can be removed through an additional vacuuming process. Thus, a vacuum is required to achieve a well-shaped microneedle. In general, in imprinting processes using polymers, gas can become trapped between the mold in the process under atmospheric pressure, forming defects, or can be trapped in the mold at the end of the pattern, causing the polymer to fail to fill the mold completely. To improve the patterning results, such cases often require performing the imprinting process in a vacuum environment. Similarly, in order to improve the sharpness of the needles, the imprinting process was performed in a vacuum chamber. In the imprinting process at an atmospheric pressure, liquid metal cannot reach the sharp parts of the PDMS mold due to the pressure of the air in normal atmosphere. However, performing this process in a vacuum environment reduces the volume of trapped air in the mold and minimizes the rebound of compressed air, making it possible to form needles with a size smaller than 10 μm, as shown in Fig. 3b.

As shown in Fig. 3c, the MAE was fabricated along with the interconnect terminal. This MAE, as shown in the magnified image, confirms that a single needle can be fabricated satisfactorily with a Bi–In–Sn alloy. To reduce electrical interference caused by contact between the skin and the microneedles, coating surfaces is necessary to block noise signals caused by sweat on the microneedles. Parylene coating is often used, but in this study a method using UV-curable PUA was proposed to simplify the fabrication process.

To minimize the residue of PUA, an appropriate amount of PUA was carefully dropped as droplets from the outside of the needles during the process, allowing PUA to permeate between the needles. After dropping the UV-curable PUA solution into the PDMS mold and pressing it with a flat PDMS, the dewetting of PUA on the PDMS surface removes most of the PUA from the needle tip, effectively reducing the remaining PUA on the metal needles. The pressed PUA forms a coating on the base plate. In addition, due to capillary action, PUA material that has risen up the sides of the needle remains after curing, as shown in Fig. 3d. As a result of the PUA coating, the needle point is exposed, and a PUA coating layer is formed on a portion of the base plate and the sides of the needle. The microneedle is not damaged in the process of coating the PUA, and the height and width after coating are reduced to 600 μm and 250 μm, respectively, resulting in a thickness of the coated PUA of about 200 μm, as shown in Fig. 3d. The length of the microneedle used in this study, 600 μm, is minimally-invasive, with a VAS of less than 1. The thickness of human stratum corneum of electrical barrier is below 50 μm and all type of microneedles are sufficient long for successful insertion into the skin layer with electrical conductivity.

### Physical tests

The strengths of the PLA microneedle and the Bi–In–Sn MAE were compared using a force displacement machine. Young's modulus of PLA is known to be in the range of several GPa. Similarly, Field's metal also has a Young's modulus value of around 8–9 GPa at a temperature of 25℃. It has been reported that Field's metal exhibits a high Young's modulus. However, the range of this modulus can vary depending on the circumstances. We therefore conducted experiments to compare the strengths of Field's metal and PLA microneedles.

To evaluate the mechanical strength of PLA microneedles and Bi–In–Sn microneedles, the force at which individual needles failed was measured. Both types of microneedles were fabricated using an identical pyramid-shaped mold measuring 340 μm wide and nearly 800 μm high. As shown in the Fig. S1, the slope of force–displacement slopes for a PLA microneedle and a Bi–In–Sn microneedle are similar, indicating that the two microneedles have similar toughness. PLA microneedles with same geometry are strong enough to penetrate skin in vivo without failure from previous study and Bi–In–Sn microneedles also have sufficient mechanical strength for insertion into the skin^35^.

**Figure 4:**
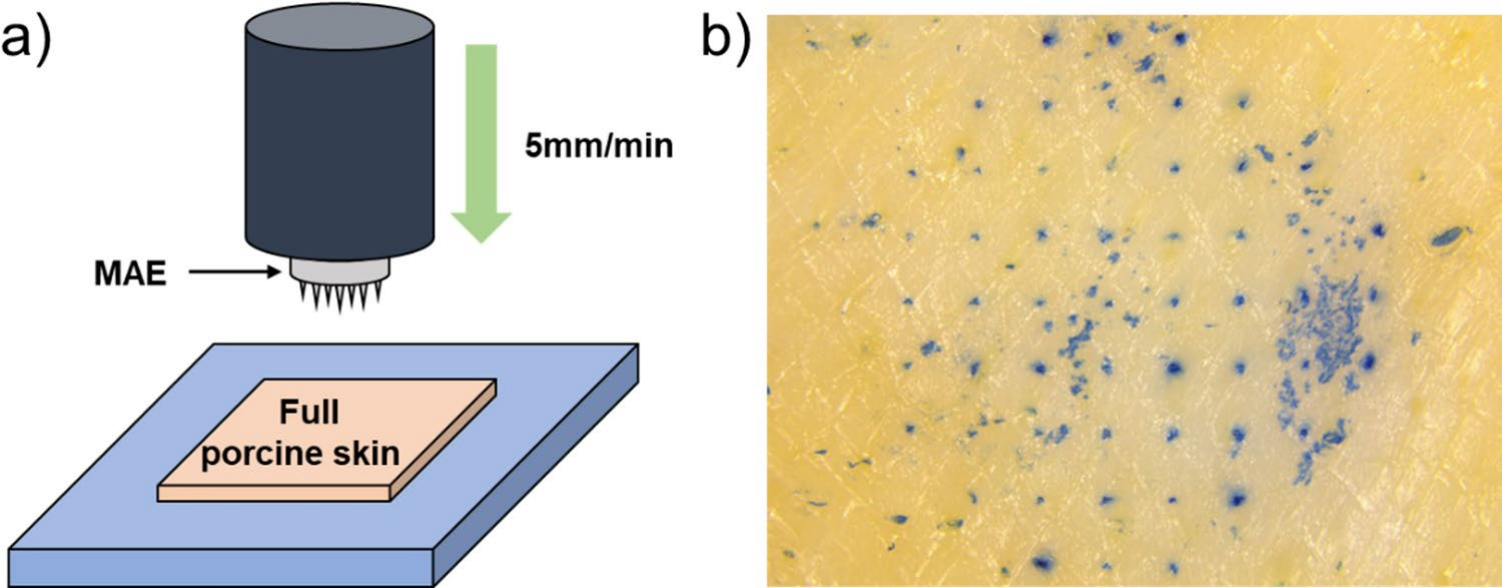
(**a**) Schematic illustration of the skin penetration performance test setup. (**b**) Stereo microscope image of full porcine skin dyed with 0.25% (v/v) trypan blue after removing the MAE.

The ability of Bi–In–Sn MAE to penetrate the skin was evaluated using a stereo microscope (TL3000 Ergo, Leica Microsystems Ltd, Switzerland); the Bi–In–Sn MAE was fabricated through the micromolding process. A schematic illustration of the machine setup is shown in Fig. 4a. As shown in Fig. 4b, out of the 57 microneedles pressed on the skin, 54 microneedles made holes that were stained on the skin surface. The successful perforation of stratum corneum was confirmed by means of trypan blue staining^36,37^. This indicates that some microneedles might not have possessed sufficient sharpness for successful penetration or were not vertically aligned during the peeling process of microneedle fabrication, resulting in their failure to penetrate. However, based on the experimental results, it can be concluded that the fabricated Bi–In–Sn microneedles demonstrate adequate strength for effective skin penetration.

### Impedance measurements

Impedance measurements were performed to evaluate the electrical performance of Bi–In–Sn MAE. When an MAE is inserted into the skin, the electrode walls come into contact with the outer skin, contributing to the impedance measurement by including the impedance of dead skin in the measured values. Therefore, it is necessary to minimize the contact between the skin and the MAE by insulating the surface of the MAE. In this study, PUA was used as the insulating layer, and UV curing was employed to fix the fluid PUA film onto the MAE surface. The impedance of the MAE was investigated by varying the thickness of the PUA film coating. The experiments utilized a 0.9% NaCl solution with a pH and conductivity similar to that of the extracellular fluid in the human body. The MAE was immersed in the 0.9% NaCl solution, and the other side was connected to the impedance measurement.

**Figure 5:**
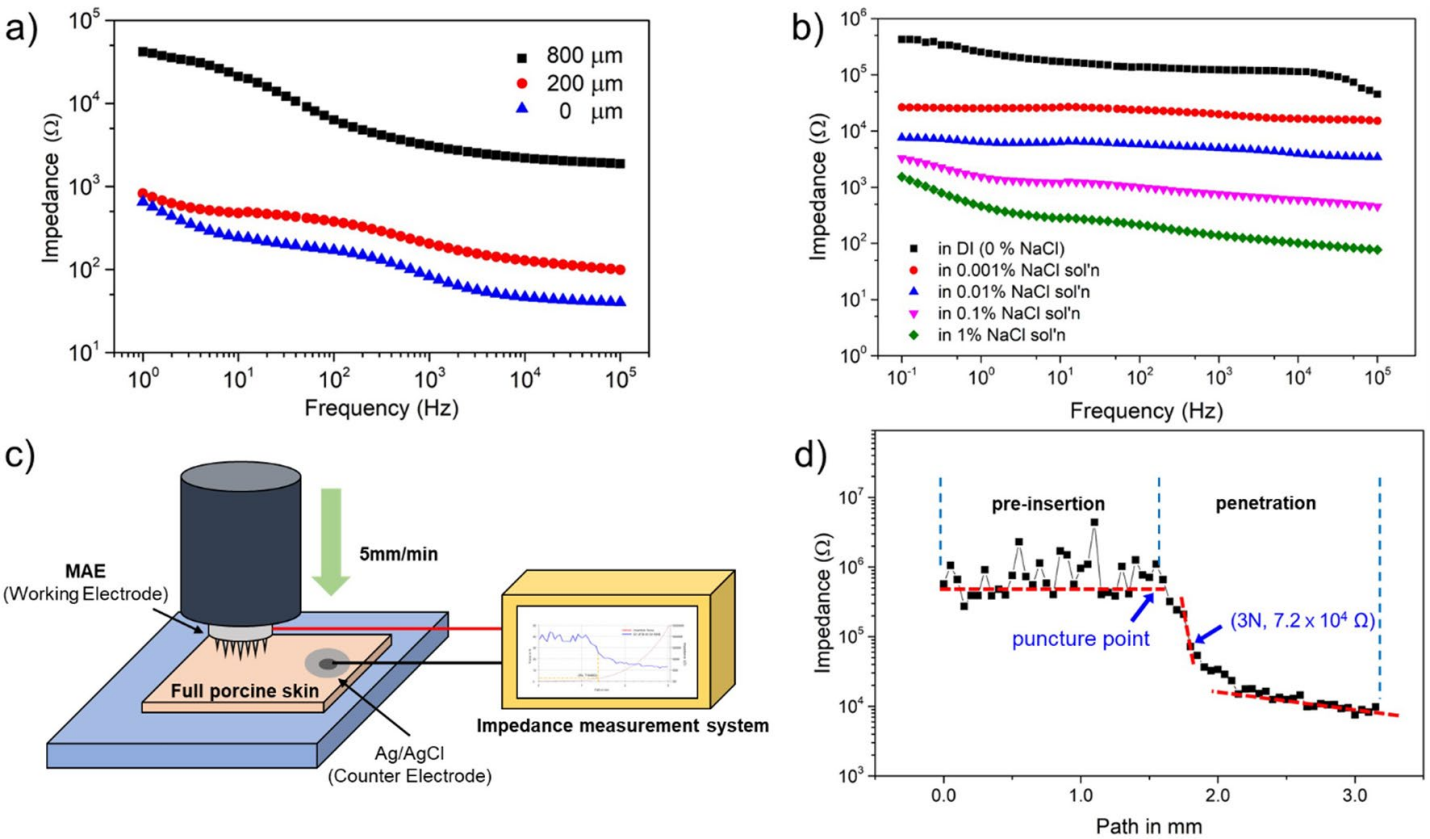
(**a**) Impedance change in 0.9% (w/v) NaCl aqueous solution according to PUA coating thickness. (**b**) Impedance change of Bi–In–Sn MAE according to NaCl electrolyte concentration from 0 to 1.0%. (**c**) Schematic illustration of the EII test setup. (**d**) Displacement-Impedance graph of Bi–In–Sn MAE.

Figure 5a presents the impedance measurements obtained by varying the thickness of the PUA coating (0, 200 μm, and 800 μm) to evaluate the insulation of the PUA film. The coating was applied to achieve a thickness of 800 μm, which closely matched the height of the microneedles with minimal tip exposure. As observed in the figure, the impedance tends to increase with thicker PUA coating, indicating improved insulation corresponding to the coating thickness. Figure 5b illustrates the impedance measurements of Bi–In–Sn MAE according to electrolyte concentration. As the NaCl electrolyte concentration increases from 0% to 1.0%, the measured impedance values exhibit a decreasing trend, as observed in the graph. Based on these results, it is evident that the PUA coating contributes to the surface insulation of the MAE.

### EII test

A schematic illustration of the EII test setup is shown in Fig. 5c. Figure 5d illustrates that the impedance, which initially remained at a high value of around hundreds of thousands of ohms before insertion, made contact with the skin when a 3N force was applied, resulting in a descent and recording of 71,848 Ω. The skin began to deform without immediately penetrating the stratum corneum due to the elasticity of the full porcine skin. Subsequently, when a force of 5N was applied, the Bi–In–Sn MAE successfully penetrated the stratum corneum, causing the impedance to drop to less than 10,000 Ω, which was maintained thereafter. Assuming all microneedles have penetrated the stratum corneum at this point, the force required for one microneedle to penetrate the stratum corneum can be calculated to be approximately 0.09 N. This force is lower than the previously measured failure force of the needle, indicating that the Bi–In–Sn MAE possesses sufficient strength to penetrate the skin.

### ECG measurement

**Figure 6:**
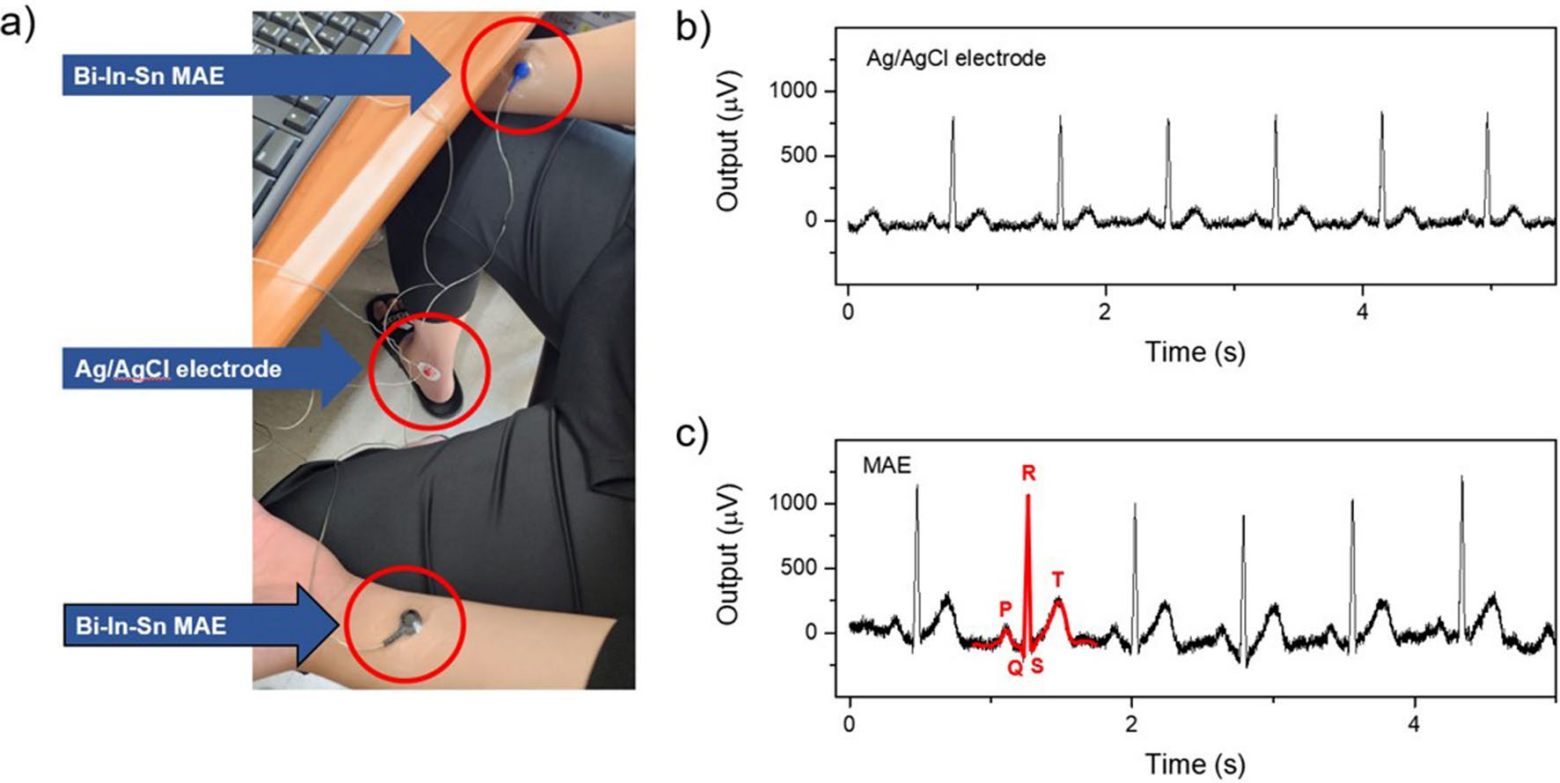
(**a**) ECG measurement image using a three-electrode method. Bi–In–Sn MAEs were attached to both wrists, and an Ag/AgCl electrode was attached to the right ankle. ECG graphs obtained using (**b**) Ag/AgCl electrodes and (**c**) Bi–In–Sn MAEs.

As shown in Fig. 6a, Bi–In–Sn MAEs were attached to both wrists of the healthy male participant, and an Ag/AgCl electrode was attached to the right ankle for measuring ECG. ECG measurements were conducted for 1 min using both Ag/AgCl electrodes and Bi–In–Sn MAEs. Figure 6b and c show a comparison of the static ECG signal recordings obtained with the wet Ag/AgCl electrode and the MAE, respectively. The raw data were then analyzed by applying a 33 Hz cut-off operation to eliminate electrode contact noise and muscle artifact noise. The Bi–In–Sn MAEs exhibited values similar to those of the Ag/AgCl electrodes. This suggests that the MAEs are capable of collecting various values due to a significant decrease in impedance. The static ECG signals recorded using both electrodes showed similar patterns, with distinguishable features shown in the figures. ECG graph obtained through the fabricated MAE clearly showed distinct P waves, QRS complexes, and T waves, as indicated by the red lines in the figure. Although there was little difference, the values obtained for the P wave, QRS complex, and T wave were slightly higher when using the MAE compared to the Ag/AgCl electrode. Taking these results into consideration, we can conclude that the performance of the fabricated MAE electrodes is comparable to that of commercial Ag/AgCl electrodes.

## Conclusions

The experiments conducted on the fabrication of a microneedle array electrode (MAE) using a Bi–In–Sn alloy have yielded significant findings. The observation of the shape of the MAEs revealed successful fabrication with minor deviations from the master mold dimensions. The use of a vacuum imprinting system during micromolding improved the sharpness and size of the microneedles, enhancing their potential for efficient, minimally-invasive skin penetration. The use of micromolding, a commercial method for manufacturing microneedles, enables the mass production of Bi–In–Sn alloy MAEs at a reasonable price. Physical tests demonstrated that Bi–In–Sn microneedles possess sufficient mechanical strength for insertion into the skin. The EII test demonstrated that Bi–In–Sn microneedles are able to penetrate the skin successfully, indicating their suitability for practical applications. The calculated force required for microneedle penetration was lower than the failure force of the needles, further supporting their effectiveness for skin insertion. Impedance measurements highlighted the importance of insulating the microneedle electrodes for optimal electrical performance. The application of PUA coating improved insulation and reduced interference, contributing to enhanced impedance characteristics. Moreover, the experiments showed that the PUA coating effectively insulated the microneedle electrodes and minimized the contact between the electrodes and skin, ensuring reliable electrical measurements. ECG measurements using the Bi–In–Sn MAEs demonstrated performance comparable to that of traditional Ag/AgCl electrodes. The recorded ECG signals exhibited distinguishable features and showed promise for accurate and reliable data collection.

Overall, these experimental results provide valuable insights into the fabrication and functionality of Bi–In–Sn microneedle arrays. The findings demonstrate their mechanical strength, successful skin penetration, improved electrical insulation, and potential applications in ECG measurements. These advancements contribute to the field of microneedle technology and pave the way for further research and development in biomedical applications, including drug delivery, diagnostics, and physiological monitoring.

### Supplementary Information

Supplementary Information.

## Data Availability

The data generated and analyzed during the current study are provided in this manuscript and can be made available from the corresponding authors upon reasonable request.
