# Supplementary material for: A study on the fabrication of metal microneedle array electrodes for ECG detection based on low melting point Bi–In–Sn alloys
Source: Sci Rep. 2023 Dec 21;13:22931. doi: 10.1038/s41598-023-50472-y (PMC10739879; doi:10.1038/s41598-023-50472-y)
Supplement: Supplementary file 1 — Supplementary Information. [file 41598_2023_50472_MOESM1_ESM.pdf]

Supplementary information

A Study on the Fabrication of Metal Microneedle Array Electrodes for ECG Detection Based on Low Melting Point Bi-In-Sn Alloys

Hyunjong Gwak[1], Sungbo Cho[2], Yoon-Jae Song[3], Jung-Hwan Park[1,*] and Soonmin Seo[1,*]

[1] Department of BioNano Technology, Gachon University, Seongnam-si, Gyeonggi-do 13120, Republic of Korea

[2] Department of Electronic Engineering, Gachon University, Seongnam-si, Gyeonggi-do 13120, Republic of Korea

[3] Department of Life Science, Gachon University, Seongnam-si, Gyeonggi-do 13120, Republic of Korea

* Corresponding authors.

E-mail addresses: pa90201@gachon.ac.kr (J.-H. Park), soonmseo@gachon.ac.kr (S. Seo),

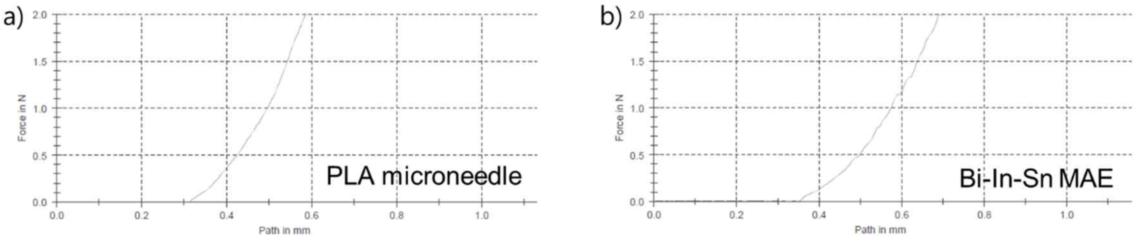

Mechanical behavior of (a) PLA microneedle and (b) Bi-In-Sn MAE

Figure S1. Mechanical behavior of (a) PLA microneedle and (b) Bi-In-Sn MAE measured using a force displacement machine.
